# Can MS lesion stages be distinguished with MRI? A postmortem MRI and histopathology study

**DOI:** 10.1007/s00415-015-7689-4

**Published:** 2015-03-13

**Authors:** Laura E. Jonkman, Alexandra Lopez Soriano, Sandra Amor, Frederik Barkhof, Paul van der Valk, Hugo Vrenken, Jeroen J. G. Geurts

**Affiliations:** 1 Department of Anatomy and Neurosciences, VU University Medical Center, Amsterdam, The Netherlands; 2 Department of Radiology and Nuclear Medicine, VU University Medical Center, Amsterdam, The Netherlands; 3 Department of Pathology, VU University Medical Center, Amsterdam, The Netherlands; 4 Department of Physics and Medical Technology, VU University Medical Center, Amsterdam, The Netherlands

**Keywords:** Multiple sclerosis, MRI, Histopathology, Postmortem, Lesions, White matter

## Abstract

In multiple sclerosis (MS), a histopathological distinction is made between different stages of white matter (WM) lesions. These lesions are characterized as preactive, active, chronic active or chronic inactive, depending on the degree of microglia activation and degree of demyelination. The different lesions are not distinguishable on conventional magnetic resonance imaging (MRI) scans at standard clinical field strengths, but might be distinguished using more advanced, quantitative MRI methods, such as T1 relaxation time (T1-RT) mapping. To investigate this, postmortem brain material from 20 MS patients was investigated, using both T1-RT MRI at 1.5T and histopathology. The brain material contained a total of 9 preactive, 18 active, 30 chronic active and 14 chronic inactive lesions, as well as 38 areas of normal appearing WM (NAWM). Our results show that, at 1.5T, T1-RT qMRI can only distinguish between categories NAWM/preactive, active and chronic WM lesions. Advanced imaging at standard field strengths, such as conventional imaging measures, is therefore insufficient to differentiate the WM lesions in MS, and higher field strengths may be required to achieve better pathological differentiation of these lesions.

## Introduction

Multiple sclerosis (MS) is an inflammatory and demyelinating disease of the central nervous system (CNS). Pathologically, a distinction can be made between different stages of white matter (WM) lesions that may be characterized as preactive, active, chronic active or chronic inactive, depending on their degree of microglia activation, adaptive immune response and demyelination [1]. Preactive lesions still have normal myelin density and morphology, but already show clusters of activated microglia. Active lesions show sharply bordered demyelination with myelin-laden macrophages. In chronic active lesions, macrophages have migrated to the rim of the lesion, leaving the center fully demyelinated and hypocellular. In chronic inactive lesions, there is complete demyelination; microglia and macrophages are no longer present. Conventional magnetic resonance imaging (MRI) techniques, such as T2-weighted MRI, are highly sensitive to MS WM lesions [2, 3] but do not provide information on the aforementioned pathological heterogeneity. However, for clinical purposes, it would be extremely useful if this pathological distinction could be made in vivo. This way, an inflammatory profile of MS patients could be more accurately described; the clinical impact of these different lesion stages, as well as their development over time, their occurrence in different patient and disease stages and their responsiveness to therapy could be monitored. Currently, the technique of choice for visualizing active inflammatory WM lesions in vivo requires the administration of intravenous contrast agents. Quantitative MRI (qMRI) techniques, such as quantitative T1 relaxation time (RT) mapping, have shown to be both sensitive and more pathologically specific [4–7]. T1-RT correlates with myelin content and axonal count, which are both decreased in lesions compared to NAWM, and axonal count differs between different lesion stages [8, 9]. T1-RT mapping should therefore have the potential to detect inflammatory lesions at earlier stages and with more subtle pathology, which are now only evident postmortem. Use of such advanced MRI techniques could then improve clinical correlations of MRI-detectable abnormalities in vivo, as these correlations are generally low when using conventional techniques [10]. Therefore, the current study used advanced postmortem MRI and histopathology correlation to investigate whether T1-RT mapping can be used to distinguish the different stages of WM demyelination in MS.

## Methods

### Patients and autopsy

Coronal brain sections of 20 patients with MS (11 females, 9 males, mean age at autopsy 63.6 ± 11.5 years, mean disease duration 26.2 ± 15.3 years) were obtained after rapid autopsy (mean postmortem delay 8 h 21 min). Table 1 provides the demographic and neuropathological details of the donors. Autopsy procedure and tissue sampling followed the MS Center Amsterdam autopsy protocol which has been described previously [11]. Briefly, for each patient, five 10-mm-thick coronal hemispheric brain sections were cut and subjected to MR imaging. WM abnormalities visible on T2-weighted imaging were sampled.

**Table 1:** Demographic and neuropathology data of patients

Patient	Sex	Age	DD (years)	PMD (h:min)	Scanner^a^	MS type	Cause of death
1	F	44	8	10:15	1	PPMS	Heart failure
2	M	63	24	7:05	1	SPMS	Cardiac arrest
3	F	69	53	7:30	1	SPMS	Heart failure
4	F	70	40	6:55	1	SPMS	Urine tract infection
5	F	57	21	20:00	1	SPMS	Decubitus
6	F	76	19	9:45	1	SPMS	Unknown
7	F	81	64	4:00	1	SPMS	Unknown
8	M	50	15	5:25	2	PPMS	Pulmonary carcinoma
9	F	66	22	6:00	2	SPMS	Unknown
10	M	49	24	8:00	2	SPMS	Pneumonia
11	F	77	24	10:00	2	SPMS	Euthanasia
12	M	72	23	7:55	2	SPMS	Pneumonia
13	M	56	14	5:00	2	SPMS	Cachexia
14	F	60	7	8:50	2	PPMS	Euthanasia
15	M	54	12	8:15	2	PPMS	Euthanasia
16	M	75	50	10:10	2	SPMS	Pneumonia
17	F	50	17	7:35	2	SPMS	Euthanasia
18	M	67	37	11:00	2	SPMS	Heart failure
19	M	54	29	7:00	2	SPMS	Euthanasia
20	F	81	21	6:30	2	PPMS	Heart failure
Mean ± SD		63.6 ± 11.5	26.2 ± 15.3	8:21			

*PMD* postmortem delay (h:min), *DD* disease duration in years since diagnosis

^a^Scanner; 1, Avanto; 2, Sonata

### MRI protocol and qMRI maps

The postmortem brain slices were scanned according to our autopsy protocol [11, 12], using a whole body 1.5T magnetic resonance system (Sonata and Avanto, Siemens Medical Systems, Erlangen, Germany) with a standard circularly polarized head coil (Sonata) or a 12-channel phased-array head coil (Avanto). Conventional Pd/T2-weighted images were acquired (TR/TE1/TE2: 2500/85/24 ms, in-plane resolution 0.5 mm × 0.5 mm, slice thickness 4 mm), centered in the middle of the slice and parallel to the coronal surface. For T1 mapping, six sets of images were acquired using a 3D gradient echo sequence (3D-FLASH; TR/TE: 20/4 ms; in-plane resolution 1 mm × 1 mm, slice thickness 4 mm), covering the same volume as the Pd/T2-weighted images. Flip angles were 2°, 5°, 10°, 15°, 20° and 25°, respectively. For B1-mapping, five sets of images were acquired (TR/TE: 20/4 ms, in-plane resolution 2 mm × 2 mm, slice thickness 4 mm). Flip angles were 140°, 160°, 180°, 200° and 220°, respectively.

### Image analysis

Pixel-by-pixel T1 calculations were performed with B_1_ correction as described by Venkatesan et al. [13]. Briefly, B_1_ maps were generated from the image volumes with nominal flip angles between 140° and 220° by determining the ratio between the true and nominal flip angle from the signal zero crossing that occurs at a true flip angle of 180°. Subsequently, T1 was determined for each pixel through a nonlinear least squares fit by using the image and the calculated B_1_ map [13].

### Histopathology and immunohistochemistry

After MRI, the tissue blocks were fixed in 10 % formalin, routinely processed and embedded in paraffin. Subsequently, 5 µm-thick sections were cut, mounted onto glass slides (Superfrost, VWR international, Leuven, Belgium) and dried overnight at 37 °C. Sections were deparaffinized in a series of xylene, 100 % ethanol, 96 % ethanol, 70 % ethanol and water. Endogenous peroxidase activity was blocked by incubating the sections in methanol with 0.3 % H_2_O_2_ for 30 min. After this, the sections were rinsed with 0.01 mol/L phosphate-buffered saline (PBS, pH 7.4). Staining and immunohistochemistry were performed on adjacent sections with antibodies against the following targets: microglia/macrophages (anti-HLA-DR, clone LN3) and proteolipid protein (PLP; Serotec, Oxford, UK). Bound primary antibodies were detected using EnVision method (DAKOCytomation, Glostrup, Denmark) and 3,3′diaminobenzidine-tetrahydrochloride dihydrate (DAB) was used as a chromogen. Sections were counterstained with hematoxylin and mounted (Depex, BDH; Poole, UK).

### Scoring, classification and matching

**Fig. 1:**
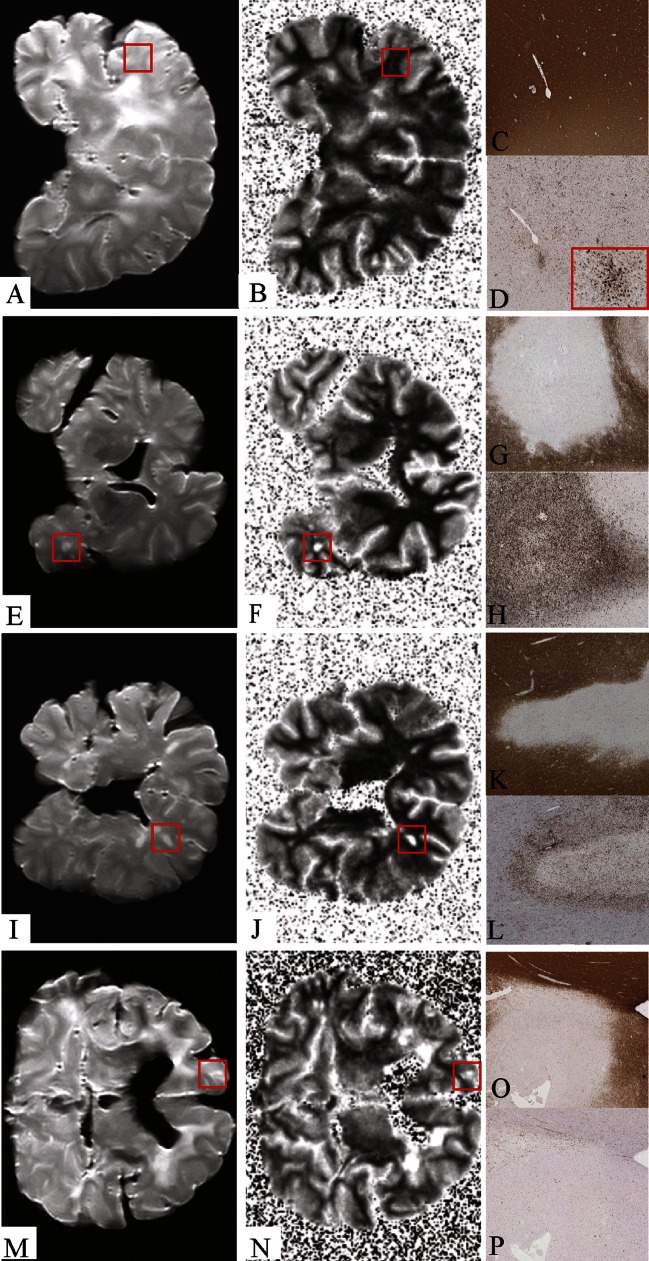
Matching of T2-w image and T1 map with histology. T2 image (**a**, **e**, **i**, **m**) and T1 map (**b**, **f**, **j**, **n**) with a *red box* indicating lesion location. A preactive lesion (**a**–**d**), an active lesion (**e**–**h**), a chronic active lesion (**i**–**l**) and a chronic inactive lesion (**m**–**p**). Lesions are visualized by histological sections of PLP (**c**, **g**, **k**, **o**) and LN3 (**d**, **h**, **l**, **p**) stainings. Note that outside the brain slice, due to the virtual absence of protons, T1-RT fits give unreliable results. However, to present un unbiased view of our analysis, we present the full data in the T1-RT images in this figure. The range for T1-RT maps is set between 500 ms (*black*) and 1,500 ms (*white*)

**Fig. 2:**
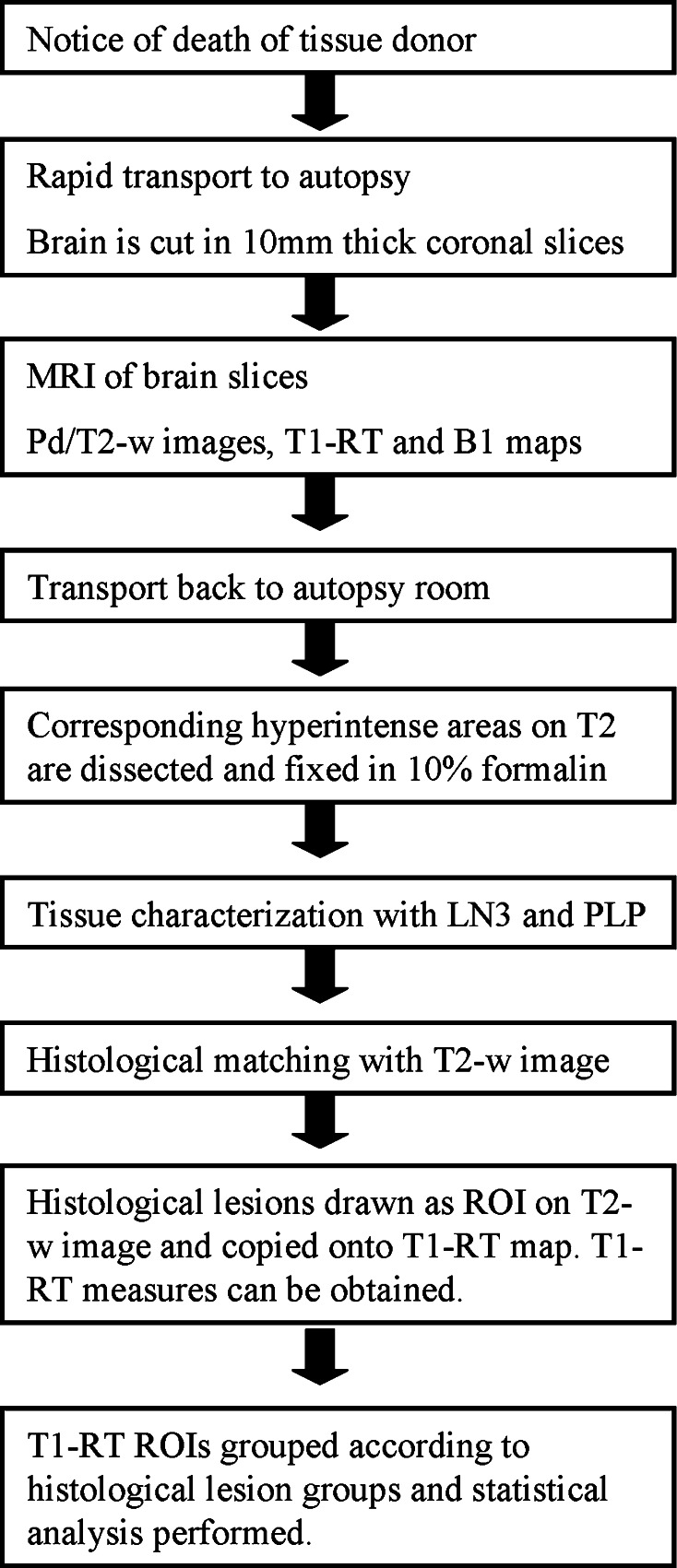
Flowchart of postmortem MRI with histology, matching and analysis

WM lesions were scored by an experienced pathological examiner (PvdV) and classified according to the van der Valk and De Groot criteria [1] into preactive, active, chronic active and chronic inactive lesions. Preactive lesions show myelin and clusters of activated microglia. Active lesions show sharply bordered demyelination with macrophages. Chronic active lesions show macrophages at the rim of the lesion, and in chronic inactive lesions microglia and macrophages are no longer present. In total, 71 WM lesions were selected: 9 preactive, 18 active, 30 chronic active and 14 chronic inactive lesions. Furthermore, 38 areas of normal appearing WM (NAWM) were selected after histopathological inspection. Sections containing WM lesions were matched to corresponding postmortem T2-weighted MR images as described previously [12]; see Fig. 1 for an example. Lesions were outlined (ROIs) on the T2 images using MIPAV software (Medical Image Processing, Analysis and Visualization, National Institutes of Health; mipav.cit.nih.gov). Subsequently, ROIs were copied onto the T1 qMRI maps and T1-RT values were obtained. ROIs were also placed in the NAWN, so as to act as a control measurement. An average T1-RT value was obtained over all voxels within a ROI (lesion or NAWM). Then each lesional/NAWM T1-RT value was assigned to their corresponding histopathological group (preactive, active, chronic active, chronic inactive or NAWM) and statistical analysis was performed. A flowchart of the autopsy, histological, matching and analyzing procedure can be found in Fig. 2.

### Statistical analysis

Descriptive and statistical analysis was performed using SPSS 20.0 for windows (SPSS, Inc., Chicago, IL). For the analysis between lesion types, we used the general estimated equation (GEE) for related data (71 lesions from 20 patients; some lesions from the same patient and therefore not independent). Scanner (Sonata or Avanto) and lesion area (mm^2^) were added as covariates. Holm–Bonferroni correction was used for multiple testing; significance level was set at *p* < 0.05.

## Results

T1-RT increased consistently when moving from NAWM through preactive and active lesions to chronic lesions. Table 2 provides an overview of the mean (±SD) T1-RT values of the different lesion types. Statistical analysis of T1-RT revealed that NAWM differed significantly from active, chronic active and chronic inactive lesion types (all *p* < 0.001). Preactive lesions also differed significantly from active, chronic active and chronic inactive lesion types (all *p* < 0.001). However, NAWM and preactive lesions did not differ significantly from each other (*p* = 0.742). Furthermore, active lesions differed significantly from chronic inactive lesions (*p* < 0.05), but not from chronic active lesions (*p* = 0.286). Chronic active and chronic inactive lesions did not differ significantly from each other (*p* = 0.316). When lesion types were grouped into three distinct groups, i.e., NAWM/preactive (mean T1-RT = 798.32 ± 67.72 ms), active (mean T1-RT = 1140.89 ± 204.82 ms) and chronic (mean T1-RT = 1271.36 ± 231.19 ms, including chronic active and chronic inactive lesions), all three groups differed significantly from each other. In other words, NAWM/preactive lesions differed significantly from active (*p* < 0.05) and chronic lesions (*p* < 0.001), and active lesions differed significantly from chronic lesions (*p* < 0.05).

**Table 2:** Mean, standard deviation and minimum and maximum of T1-RT (in ms) for lesion types

Lesion type/tissue	Mean	Standard deviation	Minimum	Maximum
NAWM^c^	785.15	78.69	704.20	981.22
Preactive^c^	846.15	66.11	744.50	939.00
Active^a,b^	1140.89	204.82	839.50	1432.45
Chronic active^a,b^	1225.58	197.77	1003.03	1561.04
Chronic inactive^a,b,d^	1320.32	370.05	942.97	2171.69

^a^Significant difference (*p* < 0.001) with normal appearing white matter (NAWM)

^b^Significant difference (*p* < 0.001) with preactive lesions

^c^Significant difference (*p* < 0.001) with active lesions

^d^Significant difference (*p* < 0.05) with active lesions

## Discussion

In the current study, we set out to investigate whether the different stages of WM demyelination that are defined in MS histopathology can be differentiated by quantitative T1-RT mapping. Upon analysis of the data, histopathologically defined WM lesion types could be distinguished from NAWM, and a distinction could also be made between the overarching categories NAWM/preactive, active and chronic lesions (including chronic active and chronic inactive lesions). However, further subclassification into pathological lesion types based on T1-RT measures was not possible.

This means that, at a standard clinical field strength of 1.5T, there is additional value in distinguishing active from chronic lesions, but determining which exact lesion types are predominant in which patients or how specific lesional stages correlate with clinical disability is still limited and requires more research, possibly at higher field strength with better signal-to-noise ratio and spatial resolution. T1-RT measurement would still be an interesting MRI candidate at higher field strength, as this technique is highly sensitive to pathology and may detect tissue abnormalities where other advanced MRI techniques cannot [4]. This remains important when attempting to visualize subtle tissue pathology such as microglial clustering or incipient demyelination. However, future studies would probably benefit from combining T1-RT with other advanced MRI measures, such as MTR or DTI, as a combination of qMRI metrics with different pathological substrates may increase pathological specificity and hence improve MRI characterization of lesional heterogeneity in MS.

A limitation of this study is that although from in vivo measurements it is known that the accuracy and reproducibility of the T1-RT mapping method used are acceptable given the field strength [4, 13], we cannot fully exclude that there may be T1-RT errors related specifically to its application in fresh brain slices. Due to the rapid autopsy setting, imaging time had to be minimized to preserve tissue. Therefore, there was no time to perform a direct quantification of any potential errors related specifically to the application in fresh brain slices by, e.g., comparing to a trusted other technique such as inversion recovery spin echo imaging with an array of inversion times, because of the long acquisition times for such techniques. Furthermore, our sample size was limited when looking at the numbers of lesions within some of the lesion categories. As a result, the power to detect differences between, e.g., preactive and other types of lesions or NAWM was a priori low. However, if lesion differentiation can be improved by future (q)MRI efforts, a translation to the in vivo setting would be highly interesting. Initially, sensitivity and specificity of classifying lesions by qMRI, or T1-RT in particular, would have to be determined. Subsequently, an in vivo study with parameters similar to those used in this postmortem study is recommended, to see how T1-RT values change between the postmortem and in vivo setting and how this affects classification. Lesional changes may then be studied in vivo and in direct relation to clinical disability, and questions regarding homo- or heterogeneity of lesional pathology within and between patients [14, 15] as well as specific responses of lesions to treatment could and should then be addressed.
